# Estimating the cost of delivering direct nutrition interventions at scale: national and subnational level insights from India

**DOI:** 10.1111/mcn.12257

**Published:** 2016-05-17

**Authors:** Purnima Menon, Christine M. McDonald, Suman Chakrabarti

**Affiliations:** ^1^ International Food Policy Research Institute (IFPRI) New Delhi India; ^2^ Famine Early Warning Systems Network (FEWS NET) Washington District of Columbia USA

**Keywords:** nutrition, health, cost, scaling up, India, South Asia

## Abstract

India's national nutrition and health programmes are largely designed to provide evidence‐based nutrition‐specific interventions, but intervention coverage is low due to a combination of implementation challenges, capacity and financing gaps. Global cost estimates for nutrition are available but national and subnational costs are not. We estimated national and subnational costs of delivering recommended nutrition‐specific interventions using the Scaling Up Nutrition (SUN) costing approach. We compared costs of delivering the SUN interventions at 100% scale with those of nationally recommended interventions. Target populations (TP) for interventions were estimated using national population and nutrition data. Unit costs (UC) were derived from programmatic data. The cost of delivering an intervention at 100% coverage was calculated as (UC*projected TP). Cost estimates varied; estimates for SUN interventions were lower than estimates for nationally recommended interventions because of differences in choice of intervention, target group or unit cost. US$5.9bn/year are required to deliver a set of nationally recommended nutrition interventions at scale in India, while US$4.2bn are required for the SUN interventions. Cash transfers (49%) and food supplements (40%) contribute most to costs of nationally recommended interventions, while food supplements to prevent and treat malnutrition contribute most to the SUN costs. We conclude that although such costing is useful to generate broad estimates, there is an urgent need for further costing studies on the true unit costs of the delivery of nutrition‐specific interventions in different local contexts to be able to project accurate national and subnational budgets for nutrition in India.

**Key messages**

- US$5.9bn/year is required to deliver 14 essential nutrition interventions at full coverage across India.
- Cash transfers to women to support breastfeeding accounts for the largest proportion of the total cost, followed by supplementary food targeted at children under two.
- The lowest cost interventions include counselling for promoting breastfeeding, iron‐folic acid supplements for pregnant and breastfeeding women, vitamin A supplementation, deworming and insecticide treated nets for pregnant women in malaria‐endemic areas.
- Scaling up costs vary considerably even within India – states in the Indo‐Gangetic area require the greatest outlay because of larger target population sizes.
- We estimate that planners can use a rule of thumb of US$140 per child 0–24 months of age per year as an average cost to budget for interventions covered in this framework but caution that more research is needed on unit costs of several interventions.

## Introduction

India is currently not on track to meet Millennium Development Goals 1 (eradicate extreme hunger and poverty) and 4 (reduce child mortality) and carries an exceptionally high proportion of the global of undernutrition. In 2005–2006, nearly half of all children under 5 years of age in India were stunted (International Institute for Population Sciences 2007). A high prevalence, coupled with a large population size, make India home to the largest number of undernourished children in the world – estimated at over 58 million in 2006. Undernutrition among women and children is determined by a diverse set of factors that include immediate, underlying and basic determinants (Black *et al.* 2013). Strategies to improve nutrition, therefore, include a set of interventions to target immediate determinants of poor diet and illnesses, typically delivered through community‐based nutrition programmes or health systems, called ‘nutrition‐specific’ interventions (Bhutta *et al.* 2013a). Interventions to strengthen the underlying determinants of food insecurity, poverty, women's status, and sanitation, called ‘nutrition‐sensitive’ interventions, are also recommended, but less evidence is available on their effectiveness (Ruel *et al.* 2013). It is, therefore, well‐accepted now that scaling up a set of nutrition‐specific interventions must be part of any strategy to combat undernutrition, while efforts continue on identifying the best combination of nutrition‐sensitive interventions for any context. In India, where nutrition policy already includes several recommended nutrition‐specific interventions, prior research has identified that the gaps to delivering nutrition‐specific interventions lie primarily in areas of implementation and monitoring (Avula *et al.* 2013). Among other actions necessary to support adequate implementation is adequate financing, and therefore, one of the critical questions that that must be asked is ‘How much will it cost?’

In public health nutrition, cost analyses are typically undertaken to offer estimates of the financial resources required to provide a service or intervention to a specific population. Costing studies help to identify the levels, types and composition of costs, as well as the overhead and infrastructure that are required to expand the coverage of an intervention. They can also isolate regions where interventions are challenging to implement and where additional resources may be required to effectively expand coverage to reach the target population. This information is critically important to programme planning and implementation. The inclusion of cost–benefit analysis can also help policymakers prioritize interventions that will have the greatest impact in situations where resources are limited (Stenberg *et al.* 2015). In addition, costing analyses aid in standardizing programme domains, accountability and incentives (Fiedler & Macdonald 2009).

In 2010, the World Bank spearheaded a study, Scaling up Nutrition: What will it Cost? (SUNWWIC) (Horton *et al.* 2010), to estimate the total cost of scaling up a package of 10 direct nutrition interventions from current coverage levels to full coverage in 36 countries that represent 90% of the global stunting burden and 32 additional smaller countries that also have high rates of child undernutrition. Following this, the second paper of the 2013 Lancet Series on Maternal and Child Nutrition provided further analyses on the cost of implementing 10 direct nutrition interventions at scale in 34 countries that carry the highest global burden of undernutrition (Bhutta *et al.* 2013b). Other authors have recently elaborately furthered the costs required for a full investment in breastfeeding promotion on a global scale (Holla *et al.* 2013). These studies all succeed in approximating the required financing to scale up important nutrition activities at the global level. They also underscore the importance of investing in nutrition and raise awareness of the need for additional resources. However, these global cost estimates do not typically capture local contexts, nuances and priorities of the individual countries. There is, therefore, a clear need for more tailored cost estimates that account for important factors such as local unit costs, synergies between interventions and optimal delivery platforms at the national and subnational level. This need is particularly pronounced in India, given its persistently high burden of undernutrition and recent findings on suboptimal coverage levels of most nutrition activities (Avula *et al.* 2013).

Within this context of costing and cost‐effectiveness in the area of nutrition, the objectives of this study are to use the SUNWWIC methodology and use local costing data and information on delivery platforms and target populations to calculate and compare the cost of delivering two sets of interventions at scale. The first is the set of the 10 SUN interventions using the most recent population data, and the second is a set of 14 nutrition interventions that are encompassed in India's policy framework and also supported by recommendations from a large network of stakeholders in India, the Coalition for Food and Nutrition Security in India (The Coalition for Sustainable Nutrition Secuirty 2010). We call this set of interventions the ‘*India Plus*’ actions. Table 1 provides a broad comparison of the two sets of interventions analysed in this paper.

**Table 1:** A comparison of the Scaling Up Nutrition (SUN) and *India Plus* interventions

SUN interventions	*India Plus* interventions
Behaviour change interventions	
Community nutrition programmes for behaviour change communication for caregivers of children 0–59 months of age	Counselling for mothers during pregnancy
	Counselling for optimal breastfeeding to caregivers of children 0–6 months
	Counselling for complementary feeding and hand washing to caregivers of children 0–6 months
Micronutrient and deworming interventions	
Vitamin A supplementation for children 6–59 months	Vitamin A supplementation for children 6–59 months
Zinc supplementation for children 6–59 months	ORS and therapeutic zinc supplements for treatment of diarrhoea for children 2–59 months
Deworming for children 12–59 months	Deworming for children 12–59 months
	Deworming for adolescents 11–18 years
Iron‐folic acid supplements for pregnant women	Iron supplements for children 6–59 months
	Iron‐folic acid supplements for adolescents 11–18 years
Iron‐folic acid supplements for pregnant and lactating women
Multiple micronutrient powders for children 6–23 months not receiving fortified food	No comparable intervention
Iron fortification of staple foods for general population	
Salt iodization for general population	
Complementary and therapeutic feeding interventions	
Complementary food for prevention or treatment of moderate malnutrition for children 6–23 months	Complementary food supplements for children 6–36 months of age
	Supplementary food rations for pregnant and lactating women for 6 months after delivery
	Additional food rations for severely malnourished (WAZ < −3) children 6–59 months
Severe Acute Malnutrition treatment	
Community‐based Management of Acute Malnutrition for children 6–59 months	Facility‐based treatment for children 6–59 months for children 6–59 months of age with WHZ < −3
Others	
No comparable intervention	Insecticide‐treated nets for pregnant women in malaria‐endemic areas
	Cash transfers to women for the first 6 months after delivery

SUN, Scaling Up Nutrition; ORS, oral rehydration salts; WAZ, Weight‐for‐Age Z score; WHZ, Weight‐for‐Height Z score.

### Aim and scope

This paper strives to estimate the costs of implementing a set of specific nutrition actions in financial or budgetary terms. It does not venture to calculate the full social resource requirements that also incorporate the opportunity costs of time committed by beneficiaries accessing the services. While this latter approach is more comprehensive, it involves the collection of primary data, which is beyond the scope of the current study. Furthermore, this paper also does not focus on cost‐effectiveness analyses or cost–benefit analyses. Rather, it focuses on providing the best possible estimates of the cost of implementing each intervention at full coverage but does not predict the corresponding health and nutrition outcomes that are expected to result from the scale up of services.

The cost estimates in this paper are restricted to direct, nutrition‐specific interventions, primarily delivered through programmes implemented by the Ministry of Health and Family Welfare and the Ministry of Women and Child Development (see Avula *et al.* 2013 for further detail) and broadly agreed upon by a national technical stakeholder coalition (Swaminathan 2009). We do not include nutrition‐sensitive interventions (e.g. nutrition‐sensitive social protection programmes, programmes to improve agricultural productivity in a nutrition‐sensitive manner or to improve sanitation). There is agreement that such interventions can help to improve nutrition outcomes in the long run, but the evidence base is weaker in comparison with nutrition‐specific interventions, delivery platforms are less clear and costing data are sparse.

This paper is a summarized version of a longer policy‐focused report on the costing of nutrition‐specific interventions in India, which is available elsewhere (Menon *et al.* 2015).

## Methods

### Costing approach

The ‘program experience’ approach is used to calculate the costs of delivering both sets of activities at full coverage (Horton *et al.* 2010). This method utilizes unit cost data for each intervention from actual programmes that are in operation and considers the context and channels through which they are delivered.

To calculate the cost of providing interventions at full coverage, we performed the following steps: (1) described each intervention to be costed; (2) defined the target population of each intervention; (3) estimated the size of the target population in 2014 for each intervention; (4) specified the platform or channel(s) through which each intervention or activity will be delivered; (5) obtained local unit cost data for *India Plus* interventions from relevant sources within India or from programmatic settings in South Asia that could be applicable; (6) for each intervention, multiplied the size of the target population by the unit cost to arrive at a total cost of implementing each intervention at full coverage; and (7) perform necessary adjustments for inflation. The Government of India has explicitly committed to ‘universalize’ the costed nutrition interventions, and therefore, we define ‘full coverage’ as 100% of the target population for all interventions except in the case of treatment of severe acute malnutrition, which we set to 80%. This is in keeping with SUNWWIC methods and is based on the reality that it is exceptionally challenging to surpass 80% coverage at scale. We first conducted all calculations at the national level and then estimated costs at the state level for all 35 Indian states and union territories using state‐specific target population estimates.

The intervention descriptions, target population and delivery channel are specified in Tables 2 and 3. Subsequently, we describe the data sources for the size of the target populations and the unit costs of interventions.

**Table 2:** Assumptions, target populations and unit costs of *India Plus* interventions

Intervention	Description	Assumptions	Target Population	Unit cost (US$)	Source
Counselling actions					
Counselling during pregnancy	Promotion of optimal nutrition during pregnancy although an average of 3.5 individual/group contacts during pregnancy	Assumes an average of 4.1 face‐to‐face visits per pregnant woman at US$0.43 per visit.	Pregnant women	$1.76 per pregnant woman per year	(Khan *et al.* 2014)
Counselling for breastfeeding	Promotion of optimal breastfeeding practices though an average of 11.7 individual/group contacts between 0–6 months of age	Assumes an average of 15.2 face‐to‐face visits between 0–6 months at US$0.11 per visit.	Caregivers of children 0–6 months of age	$1.67 per child 0–6 months of age per year	(Khan *et al.* 2014)
Counselling for complementary feeding and hand washing	Promotion of optimal IYCF and hand‐washing practices through an average of 11.6 individual/group contacts between 6–12 months of age, and 13.5 contacts between 12–24 months of age	Assumes an average of 13.3 face‐to‐face visits per child between 6–12 months of age at US$0.56 per visit, and an average of 12.2 face‐to‐face visits per child between 12–24 months of age at US$0.23 per visit.	Caregivers of children 6–24 months of age	$7.47 per child 6–12 months of age per year $2.80 per child 12–24 months of age per year	(Khan *et al.* 2014)
Supplementation					
Complementary food supplements	Daily food supplements between 6–36 months of age	Assumes provision of a daily ration at Rs.6 (US$0.097) per day.	Children 6–36 months of age	$14.52 per child 6–12 months of age per year $29.03 per child 12–26 months of age per year	(Ministry of Women and Child Development 2012)
Supplementary food rations	Daily food supplements for the second and third trimesters (i.e. approx. 6 months) of pregnancy and the first 6 months of lactation	Assumes provision of a daily ration for 6 months during pregnancy and 6 months after birth at Rs.7 (US$0.11) per day.	Pregnant and lactating women for 6 months after delivery	$16.93 per pregnant woman per year; $16.93 per mother of a child 0–6 months of age per year	(Ministry of Women and Child Development 2012)
Additional food rations for severely malnourished children	Provision of an additional daily food supplement for 3 months for children who are severely malnourished	Assumes provision of a daily ration for 3 months at Rs.9 (US$0.145) per day.	Children 6–59 months of age with WAZ < −3	$13.06 per severely underweight child 6–36 months of age per year	(Ministry of Women and Child Development 2012)
Micronutrient and deworming					
IFA supplements for pregnant and breastfeeding women	Provision of IFA supplements for women	Provision of daily IFA supplements for women during the second and third trimesters of pregnancy and for 6 months after delivery	Pregnant and lactating women for 6 months after delivery	$0.72 per pregnant woman per year; $0.51 per mother of a child 0–6 months of age per year	(Micronutrient Initiative 2011)
IFA supplements and deworming for adolescents	Provision of IFA supplements through the school system	Assumes weekly provision of IFA tablets and semi‐annual deworming prophylaxis	Adolescents 11–18 years of age	$0.40 per adolescent 11–18 years of age per year	(UNICEF 2011)
Iron supplements for children	Provision of daily iron supplements for children 6–59 months of age	This is the GOI's current expenditure on iron supplementation per beneficiary	Children 6–59 months of age	$0.37 per child 6–36 months of age per year	(Micronutrient Initiative 2011)
Vitamin A	Supplements for children	Assumes two rounds of vitamin A supplementation per child per year	Children 6–59 months of age	$0.07 per child 6–59 months of age per year	(Micronutrient Initiative 2011)
ORS and therapeutic zinc supplements for treatment of diarrhoea	Daily ORS and zinc for 14 days during/following an episode of diarrhoea	Assumes each child 2–59 months of age has an average of three episodes of diarrhoea per year, two ORS sachets are required to treat each episode of diarrhoea, zinc is provided for 14 days per episode	Children 2–59 months of age with diarrhoea	$0.64 per child 2–59 months of age per year	(Micronutrient Initiative 2011)
Deworming	Deworming tablets for children	Assumes two rounds of deworming per child per year	Children 12–59 months of age	$0.23 per child 12–59 months of age per year	(Ministry of Health and Family Welfare 2012)
Health interventions					
Treatment of severe acute malnutrition	Facility‐based treatment for children with severe acute malnutrition	Assumes that the incident cases of SAM per year is twice the prevalence of severe wasting; 15% of these children will receive inpatient treatment; average duration of treatment is 12.5 days	Children 6–59 months of age with a WHZ < −3	$107.38 per case treated per year	(Ministry of Health and Family Welfare 2011)
Insecticide‐treated nets	Provision of insecticide treated bed nets to pregnant women for prevention of malaria in malaria‐endemic areas	Endemic areas include: Chhattisgarh, Jharkhand, Odisha, West Bengal, Arunachal Pradesh, Assam, Manipur, Meghalaya, Mizoram, Nagaland and Tripura; and Andaman and Nicobar Islands	Pregnant women	$4.84 per pregnant woman per year	(UNICEF 2013)
Miscellaneous interventions					
Cash transfers to women	Monthly cash stipend provided to breastfeeding mothers	Includes the cost of the benefit and incentives. The benefit is provided for 6 months after delivery. Excludes women working in the government sector per year	Breastfeeding mothers for the first 6 months after delivery	$103.22 per eligible woman	(Ministry of Law and Justice 2013)

IYCF, infant and young child feeding; IFA, Iron‐folic acid; GOI, Government of India; ORS, oral rehydration salts.

**Table 3:** Total costs of delivering *Scaling Up Nutrition Interventions* actions at scale across India

Intervention	Assumptions	Unit cost (US$)	Cost (US$ million) per year	Share in cost (%)
Community nutrition programmes for behaviour change communication	Assumes two children under 5 per household	$15.00 per household per year (or $7.50 per child under 5 years of age)	891.42	21.11
Vitamin A supplementation	Assumes two doses per year	$1.20 per child 6–59 months of age per year	129.79	3.07
Zinc supplementation	Allows for two to three rounds of zinc supplementation per child per year	$1.00 per child 6–59 months of age per year	5.54	0.13
Multiple micronutrient powders	Assumes each child will receive 60 sachets. Target population does not include children receiving complementary food for the prevention of moderate malnutrition.	$3.60 per child 6–23 months of age per year	4.84	0.11
Deworming	Assumes two rounds per year	$0.50 per child 12–59 months of age per year	59.43	1.41
IFA supplements	Assumes that pregnant women will receive IFA supplements for the last two trimesters of pregnancy.	$2.00 per pregnancy	56.37	1.33
Iron fortification of staple foods	General population	$0.20 per person per year	255.07	6.04
Salt iodization	General population	$0.05 per person per year	63.77	1.51
Complementary food for prevention or treatment of moderate malnutrition	Assumes ~ 250 kcal/day should be provided to each targeted child on a daily basis, because the prevalence of wasting (WHZ < −2) is > 10%	$51.10 per child per year	1649.4	39.06
Treatment of SAM using a Community‐based Management of Acute Malnutrition	Prevalence of severe wasting is doubled to estimate the incidence of SAM cases over a one‐year period. Assumes that if all other interventions are delivered first, the prevalence of SAM will decrease by 50%. Full coverage is then defined as 80% of this remainder.	$200 per child treated	1107.51	26.22
All SUN interventions			4223.14	100

IFA, Iron‐folic acid; SAM, severe acute malnutrition; SUN, Scaling Up Nutrition; WHZ, Weight‐for‐Height Z score.

### Data sources

1. Target populations: We used India's 2011 Census and accompanying Sample Registration System as the main source of data for estimating the size of each target population in 2014, as it is the most credible source of demographic information in the country. 1Available at http://www.censusindia.gov.in/2011census/population_enumeration.html (Accessed 19^th^ March 2015) More specifically, we used data on the aggregated population, age‐specific strata for males and females, the crude birth rate and the derived average population growth rate that is reported in the Sample Registration System bulletins and vital statistics sections by the Ministry of Home Affairs. 2Available at http://www.censusindia.gov.in/2011-common/Sample_Registration_System.html (Accessed 19^th^ March 2015) Our secondary data source was the third series of the National Family Health Survey, which we used to derive estimates of the prevalence of stunting, wasting, underweight, severe wasting and severe underweight among children under 5 years of age (International Institute for Population Sciences 2007). Finally, we used data from the 68th round of the National Sample Survey on employment and unemployment to estimate the percentage of women aged 18–50 years who work in the government sector. 3Available at http://mospi.nic.in/Mospi_New/site/inner.aspx?status=3&menu_id=31 (Accessed 19^th^ March 2015) The sources of data for the target population estimates for the SUNWWIC and *India‐Plus* were the same, but because target populations vary between the two sets of interventions, they were estimated appropriate to the intervention.
2. Unit costs: In performing the analyses to estimate the SUN costs, we used the same unit costs as for the 10 core SUN interventions used in SUNWWIC (Horton *et al.* 2010). For the *India Plus* interventions, we estimated local unit costs from a variety of sources. These are described subsequently.
  - Interpersonal counselling for behaviour change: There are no detailed, high‐quality costing studies on successful nutrition behaviour change communication programmes in India. Therefore, our unit cost estimates for the counselling activities were based on a recent study that estimated the implementation costs of the Alive and Thrive (A&T) initiative in Bangladesh (Khan *et al.* 2014). A&T aims to improve infant and young child feeding practices at scale through the use of intensive community‐based interpersonal counselling and national media campaigns. The authors of the costing study calculated costs per visit for the face‐to‐face interpersonal counselling sessions, which includes the costs of staff, logistics and supplies, travel, incentives, monitoring and materials. We multiplied this cost per visit by the estimated number of visits each beneficiary would receive per year to arrive at the total annual cost per beneficiary of counselling during pregnancy, counselling for breastfeeding and counselling for complementary feeding and hand washing. We note that the delivery platform in the case of the A&T initiative in Bangladesh is very similar to existing government community health outreach platforms in India.
  - Supplementary food: We used the Ministry of Women and Child Development's 2013 revised norms for the supplementary nutrition components of the Integrated Child Development Services (ICDS) programme to estimate the cost per beneficiary for supplementary food rations for children 6–36 months of age, pregnant and lactating women and severely malnourished children (Ministry of Women and Child Development 2012). There are currently no clear estimates of the actual costs of producing, delivering and promoting the consumption of high‐quality supplementary foods in the Indian context or in South Asia.
  - Micronutrient supplementation and other commodities: Estimates of the unit costs of iron‐folic acid (IFA) supplements for pregnant women, iron supplementation for children, vitamin A supplementation for children and therapeutic zinc supplements were based on detailed unit cost estimates provided in the Micronutrient Initiative's 2007–2011 National Micronutrient Investment Plan for India (Micronutrient Initiative 2011). These estimates include the costs of physical inputs as well as the delivery costs, including training, information, education and communication materials, and programme monitoring and evaluation. The combined unit cost of weekly IFA supplements and semi‐annual deworming prophylaxis for adolescents was obtained from a 2011 report by UNICEF India titled The Adolescent Girls Anaemia Control Program: Breaking the Inter‐Generational Cycle of Undernutrition in India with a focus on Adolescent Girls (UNICEF 2011). The unit cost of providing two rounds of deworming to children 12–59 months of age was calculated from data in India's National Rural Health Mission's Project Implementation Plan (Ministry of Health and Family Welfare 2012). We also used the National Rural Health Mission's Project Implementation Plan to obtain unit cost estimates of oral rehydration salts and assumed that each child 2–59 months of age would have an average of three episodes of diarrhoea per year. The estimated cost of an insecticide treated bed net was provided by UNICEF (UNICEF 2013).
  - Treating severe acute malnutrition: We estimated the per beneficiary cost of facility‐based treatment of severe acute malnutrition using the Ministry of Health and Family Welfare's 2011 Operational Guidelines and assumed an average stay of 12.4 days in the treatment facility (Ministry of Health and Family Welfare 2011). India does not currently have guidelines for community‐based management of acute malnutrition, and thus, unit cost estimates can only be derived for facility‐based treatment.
  - Cash transfers to women in the first 6 months after delivery: India's 2013 Food Security Bill (Ministry of Law and Justice 2013) currently includes a ‘maternity benefit’ for breastfeeding mothers, which is a cash transfer to women for the first 6 months after the delivery of an infant. It is targeted to those who are not employed in government, because maternity leave benefits for government employees is already in place. Receipt of the cash transfer is conditional on fulfilling the use of basic health care and breastfeeding exclusively. Although this programme has not been evaluated for impact on health outcomes, it was included in the costing exercise given prior literature on the potential for conditional cash transfer programmes to help support nutrition improvements (Ruel *et al.* 2013) and the inclusion of this intervention in India's policy framework

All unit cost estimates, the source of data, and relevant assumptions for the *India Plus* interventions are summarized in Table 2.

## Results

**Figure 1:**
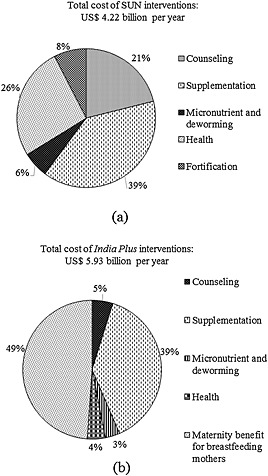
Comparison of the cost components of delivering Scaling Up Nutrition (SUN) or *India Plus* interventions at scale in India. In Panel (a) ‘Counselling’ includes community nutrition programmes for behaviour change communication; ‘Supplementation’ includes complementary food for prevention or treatment of moderate malnutrition; ‘Micronutrient and deworming’ includes vitamin A supplementation, zinc supplementation, multiple micronutrient powders, deworming, iron‐folic acid (IFA) supplements; ‘Health’ includes treatment of severe acute malnutrition using community‐based management of acute malnutrition and ‘Fortification’ includes iron fortification of staple foods and salt iodization. In Panel (b) ‘Counselling’ includes counselling during pregnancy, counselling for breastfeeding, counselling for complementary feeding and hand washing; ‘Supplementation’ includes supplementary food rations for pregnant and lactating women, complementary food supplements for children 6–36 months of age, additional food supplements for severely malnourished children; ‘Micronutrient and deworming’ includes IFA supplements for pregnant and breastfeeding women, IFA supplements and deworming for adolescents, iron supplements for children 6–36 months of age, vitamin A supplementation, oral rehydration salts and therapeutic zinc supplements for treatment of diarrhoea and deworming; ‘Health’ includes facility‐based treatment for severe acute malnutrition and provision of insecticide treated nets for pregnant women in malaria‐endemic areas; ‘Maternity benefit for breastfeeding mothers’ refers to cash transfers to women for the first 6 months after delivery.

The total annual cost of implementing the 10 core SUN interventions at full coverage, nationwide, was estimated to be US$4.22bn (Table 3). The total annual cost of implementing the complete set of *India Plus* interventions at full coverage throughout India is US$5.93bn (Table 4). The largest proportion of the total *India Plus* cost, approximately US$2.9bn and US$2.3bn, is for the cash transfers to women to support breastfeeding and supplementary food rations, respectively; these two costs together cover >80% of the total cost estimates. This is followed by health interventions (including inpatient treatment of severe acute malnutrition), counselling actions and micronutrient supplements and deworming, which account for the 4, 5 and 3% share of the total cost, respectively. Comparisons between costs of the SUN interventions and *India Plus* interventions are shown in Fig. 1; they illustrate that the SUN interventions cost more than the *India Plus* actions for all of the four comparable categories stated in Table 1 but that *India Plus* costs more for the supplementary food interventions.

**Table 4:** Total costs of delivering *India Plus* actions at scale across India

Action	Cost (US$ million) per year	Share in cost (%)
Counselling		
Counselling during pregnancy	49.61	0.84
Counselling for breastfeeding	17.87	0.30
Counselling for complementary feeding and hand washing	219.56	3.70
Supplementation		
Complementary food supplements for children 6–36 months of age	1526.01	25.73
Supplementary food rations for pregnant and lactating women	658.35	11.10
Additional food rations for severely malnourished children	111.04	1.87
Micronutrient and deworming		
Iron‐folic acid supplements for pregnant and breastfeeding women	19.83	0.33
IFA supplements and deworming for adolescents	40.19	0.68
Iron supplements for children 6–36 months of age	40.02	0.67
Vitamin A supplementation	7.57	0.13
ORS and therapeutic zinc supplements for treatment of diarrhoea	70.99	1.20
Deworming	22.41	0.38
Health		
Treatment of severe acute malnutrition	222.98	3.76
Insecticide treated nets for pregnant women in malaria‐endemic areas	24.76	0.42
Miscellaneous		
Cash transfers to women in the first 6 months after delivery	2899.73	48.89
Total	5930.91	100.00

There is considerable variability in the costs for delivering the *India Plus* interventions at scale in the different states across India (Table 5), with variability in cost estimates primarily driven by differences in target populations. The cost of implementing all *India Plus* interventions in the state of Uttar Pradesh will amount to just under of US$1.2bn, which is 20% of the total *India Plus* cost estimate. Costs for Uttar Pradesh are driven up primarily by the existing population and high fertility rates as well as by the state's poor performance on nutrition, which amplifies the costs for treatment of severe acute malnutrition. Similarly, in other states such as Bihar, Madhya Pradesh, Rajasthan and Maharashtra where wasting rates and population sizes are high, delivering interventions at scale will cost in excess of US$400m per year.

**Table 5:** State‐wise costs of *India Plus* actions at scale

Million	Total population (person)	Counselling (US$)	Supplementation (US$)	Micronutrient and deworming (US$)	Health (US$)	Cash transfers to women (US$)
Indo‐gangetic plains (Subtotal)	427.7	108.3	903.9	90.6	102.1	1197.1
Uttar Pradesh	199.6	51.4	420.8	42.7	32.5	616.9
Bihar	103.8	30.1	266.5	25.5	33.9	324.0
West Bengal	91.3	17.7	140.7	15.0	17.8	164.2
Jharkhand	33.0	9.0	75.8	7.4	17.9	91.9
Central states (Subtotal)	252.5	60.8	475.2	46.7	62.0	587.1
Madhya Pradesh	72.6	20.1	162.4	15.3	29.2	216.8
Maharashtra	112.4	24.3	186.2	18.7	14.9	207.2
Chhattisgarh	25.5	6.9	52.8	5.2	7.8	70.9
Odisha	41.9	9.5	73.8	7.4	10.1	92.2
Western (Subtotal)	182.1	45.3	356.9	35.1	31.4	454.8
Rajasthan	68.6	19.2	152.6	14.9	16.6	200.6
Gujarat	60.4	14.0	113.8	11.1	9.8	143.3
Haryana	25.4	6.5	49.0	4.8	3.6	61.6
Punjab	27.7	5.6	41.6	4.3	1.4	49.3
Southern (Subtotal)	252.8	50.0	375.7	38.8	33.2	464.5
Andhra Pradesh	84.7	16.6	123.1	12.9	6.6	160.5
Karnataka	61.1	13.1	101.7	10.2	9.1	126.8
Tamil Nadu	72.1	13.8	103.1	10.7	14.3	123.8
Kerala	33.4	6.3	45.7	4.8	3.0	51.3
Goa	1.5	0.3	2.0	0.2	0.2	2.0
Northern (Subtotal)	29.5	7.4	56.2	5.8	4.5	58.8
Jammu and Kashmir	12.5	3.6	26.9	2.8	1.9	25.2
Uttaranchal	10.1	2.4	18.4	1.9	1.5	21.3
Himachal Pradesh	6.9	1.4	10.9	1.1	1.1	12.3
North eastern (Subtotal)	44.5	11.2	90.0	9.1	13.1	104.2
Meghalaya	3.0	1.1	8.6	0.8	2.9	8.0
Tripura	3.7	0.8	6.1	0.6	1.1	5.9
Manipur	2.7	0.6	4.5	0.5	0.4	4.4
Nagaland	2.0	0.4	3.5	0.4	0.5	3.4
Arunachal Pradesh	1.4	0.3	2.9	0.3	0.4	3.1
Assam	31.2	7.9	63.7	6.4	7.7	78.2
Sikkim	0.6	0.1	0.8	0.1	0.1	1.1
Union territories (Subtotal)	21.2	4.6	35.3	3.6	3.8	41.0
Delhi	16.8	3.6	27.4	2.8	3.0	32.5
Puducherry	1.2	0.3	2.0	0.2	0.2	2.3
Mizoram	1.1	0.3	2.4	0.2	0.2	2.0
Chandigarh	1.1	0.2	1.6	0.2	0.2	1.8
Dadra and Nagar Haveli	0.3	0.1	0.9	0.1	0.1	1.1
Andaman and Nicobar Islands	0.4	0.1	0.6	0.1	0.1	0.6
Daman and Diu	0.2	0.1	0.4	0.0	0.0	0.5
Lakshadweep	0.1	0.0	0.1	0.0	0.0	0.1

Finally, our estimates for *India Plus* costs lead to an average estimated cost per child (0–24 months) per year of US$54.2 for food supplements, US$68.4 for cash transfers, US$6.8 for a full package of counselling, US$4.7 for micronutrient supplementation and deworming, US$5.9 for health interventions (excluding immunizations). This leads to a cost of US$140 per child per year.

## Discussion

In this costing exercise, we set out to estimate a set of costs for delivering at scale a range of preventive, promotive and therapeutic interventions for nutrition in India's diverse landscape. Using the SUNWWIC unit costs and India‐specific target populations, we estimated that about US$4.2bn would be needed to deliver at scale the SUN interventions in India. Using a more tailored, but expanded, set of interventions already in India's policy landscape and a set of unit costs tailored to the Indian/South Asian context, we find that costs would be about US$5.9bn cost for the set of actions we labelled ‘*India Plus*’. We find that the costs and the differences in total costs between the two methods vary depending on the interventions chosen, unit costs and target populations. We only estimated state‐specific costs for the *India Plus* set of interventions and find there that the costs are driven both by population size and the levels of undernutrition in each state. Costs are highest for Uttar Pradesh, followed by Bihar, Maharashtra, Rajasthan and other states. For the *India Plus* interventions, our findings indicate that the supplementary food and cash transfers to women together account for over 80% of the total estimated costs.

Overall, the costs estimated in this paper tally reasonably well with estimates from previous reviews and studies. For instance, in SUNWWIC, the World Bank (Horton *et al.* 2010) estimates that the total additional costs of all 10 SUN interventions is about US$5.9bn for South Asia (Afghanistan, Bangladesh, India, Nepal and Pakistan), and in the Lancet, (Bhutta *et al.* 2013a) the figure estimated is US$4.8bn.

While major studies at the global level (Horton *et al.* 2010; Bhutta *et al.* 2013b; Darmstadt *et al.* 2008) focused on providing costs for multiple interventions for South Asia as a whole, other focused studies (Fiedler & Macdonald 2009; Neidecker‐Gonzales *et al.* 2007; Bhutta *et al.* 2013a) have provided country‐specific costs for micronutrients, behavioural change communication, vaccination and fortification. To our knowledge, this study is the first to have estimated costs for multiple interventions at the *subnational* level.

### Sensitivity of estimates to unit costs and target populations

Cost estimates are highly sensitive to unit costs, which are highest for food supplementation and cash benefits. As with other studies, our estimates reaffirm that unit costs for micronutrients and deworming are lowest among the spectrum of interventions, and therefore, yield the lowest total intervention costs. Nevertheless, even unit costs can vary across countries and within, and total costs can therefore be sensitive to this variability. For example, Nepal's National Vitamin A programme reports a unit cost of US$0.04 per capsule, which is US$0.03 less than the unit cost used in this study but excludes the costs of training, personnel and promotion (Neidecker‐Gonzales *et al.* 2007). Adding in those costs increases the unit cost to US$0.82. In another example, the SUNWWIC (Horton e*t al.* 2010) estimate for unit costs for counselling is US$7.5 per child per year on average, while we used unit costs of US$1.76 for pregnancy‐related counselling, 1.67 for breastfeeding counselling (0–6 months), 7.47 for complementary feeding counselling (6–12 months) and 2.8 for counselling between 12–24 months, yielding a total cost that is lower than the SUNWWIC estimate. Another study on the costs of providing counselling have used a slightly higher unit cost than SUNWWIC on account of factoring in an additional cost to training workers of US$0.20 per child per year (Holla *et al.* 2012). We believe the unit costs applied in our study are likely the most applicable for the South Asian context as they draw on a detailed costing study that assesses the financial and economic costs of delivering a package of counselling services in a delivery platform that is similar to health systems in South Asia.

One of the most challenging areas for estimating unit costs is the cost of delivering a high‐quality nutritional supplement as part of the supplementary nutrition programme. Global recommendations for interventions support the inclusion of a food supplement or cash transfer along with counselling for behaviour change (Bhutta *et al.* 2013b). However, the cost of providing a high‐quality supplementary food is not well‐studied. Cost estimates for South Asian countries in SUNWWIC are based on a complementary food developed by the World Food Program (called India ready‐to‐use food) at US$0.13 per child per day (Horton *et al.* 2010), whereas *India Plus* estimates are based on cost norms of US$0.097 per child per day for the ICDS supplementary nutrition programme, as budgeted by the government of India. In the context of the *India Plus* estimates, we chose to use the government of India's stated cost norms for supplementary food in the ICDS programme. We recognize, however, that the cost norm of US$0.097 (INR 6) per child per day (Ministry of Women and Child Development 2012) may be unlikely to deliver a high‐quality supplementary food that also meets available guidance on the quality of supplementary foods for complementary feeding. The government of India cost norms for supplementary nutrition aim to deliver 500 kcal in calories and 12–15 grammes in protein, for 300 days a year, to children 6–36 months, at US$29 per beneficiary per year through the ICDS programme. The SUNWWIC complementary food supplements cost a total of US$51.1 per child per year to provide 260 kcal (per day) to moderately malnourished children in India. It would be prudent, given the variability in what the current cost norms are likely to be able to deliver across India, for a careful review of the composition, quality and nutritional appropriateness of the supplementary foods intended to be provided in India. Further research on the true unit costs of provision of a palatable, safe, high‐quality food supplement in India and other South Asian countries is thus strongly merited.

In the *India Plus* estimates, complementary food supplements, even using the slightly lower cost norms as noted above, will cost US$1.5bn per year. The internationally comparable intervention in the SUNWWIC costing is ‘complementary food for prevention or treatment of moderate malnutrition’. One major area of difference between the SUNWWIC and the *India Plus* estimates we derived is that the ICDS targets all children aged 6–36 months for food supplements irrespective of their nutritional status, whereas SUN interventions are targeted to children 6–23 months with a weight‐for‐age z‐scores of less than −2. This leads to differences between the two costing approaches because of target population definitions. The target population for the SUN intervention is narrower and hence smaller than the universal age‐based targeting in the ICDS programme. Research in other contexts suggests that a blanket age‐targeted programme for supplementary food is likely to have greater community‐wide impacts on undernutrition (Ruel *et al.* 2007). Even though the SUNWWIC intervention accommodates for targeting errors by assuming twice the prevalence of weight‐for‐age z‐scores < −2, the resultant target populations using the SUNWWIC and *India Plus* methods are 32.2 and 57.9 million children, respectively. These vastly different target populations yield different total costs depending on the unit cost applied. If one applies the US$29 *India Plus* unit cost to the SUNWWIC target population, the total cost is approximately US$0.93bn, which is much less than the US$1.65bn figure using the SUNWWIC unit cost. On the other hand, applying the SUNWWIC cost of supplementary food ($0.13 per child per day) would lead to a total higher cost of US$2.96bn for the *India Plus* estimate given the different target groups.

Our estimates suggest that, at US$2.9bn per year, the universally targeted cash transfers to women to support breastfeeding are the highest cost intervention to deliver at scale. These estimates too are subject to unit cost and target population variability, however. For example, one recent estimate in India (Holla *et al.* 2012) suggests that delivering cash transfers of US$2 per day for 6 months to a target population of women from households living below the poverty line in South Asia would cost US$4.8bn a year. A key difference between this estimate and what is currently budgeted in the government norms for maternity benefits is the unit cost – US$360 per woman for the Holla *et al.* estimate compared with about US$100 per woman. In this particular example, either a small increase in per day transfers for a universal intervention or a much higher transfer amount for a more targeted intervention will both have significant implications for total financial outlays.

### Limitations

Our approach to deriving estimates of the total cost of delivering nutrition‐specific interventions in India is not without some limitations. Although there will likely be differences in costs of delivery between and within different states, the lack of detailed costing studies precludes an accounting for local unit cost variations in our state‐specific estimates. Key factors that influence the cost of delivery and likely vary by state include: the level of existing infrastructure, the quality and effectiveness of existing delivery platforms, population density, the target population's accessibility to and utilization of delivery platforms and the potential need for outreach programmes. For example, interstate variations in delivering a package of IFA supplements, deworming tablets and nutrition counselling as part of the adolescent anaemia programme ranged from US$0.11 per girl per year in Tamil Nadu vs. US$0.58 in Rajasthan (UNICEF 2011). Furthermore, some of our unit costs are based on relatively small programmes in comparison with the scale of operations in India, especially in some of the larger states within India. Our analyses assume constant economies of scale in expanding the coverage of these; however, in reality, there are likely to be cost savings when implemented on a large scale. In this paper, we also do not attempt to estimate gaps between projected costs and actual expenditures, primarily because actual expenditures are difficult to track for all essential nutrition interventions.

Another limitation for interpreting the estimates derived here is data availability for the target population estimates used in deriving costs of treatment for severe acute malnutrition. The primary source of data for nutrition indicators is the National Family Health Survey (International Institute for Population Sciences 2007) from 2005–2006, which is now outdated by 10 years. Recent estimates, only provisionally released by the government of India (Ministry of Women and Child Development 2015), suggest that wasting rates in India may well have gone down by several percentage points, which would, in turn, lead to significant reductions in the numbers of severely malnourished children (International Food Policy Research Insitute 2014). This will have significant financial implications for the costing of treatment of severe acute malnutrition, one of the more expensive interventions.

Another limitation of our estimates are that we have not accounted for the cost of formative research or mass media campaigns for behaviour change communications to promote appropriate infant and young child feeding practices. The literature suggests that the costs of mass media can be quite high (US$1–5 per beneficiary at 1992 prices) and could likely increase behavioural change communication outlays considerably (Horton 1992). However, these costs will need to be estimated either at the state level or the regional level, given the diversity across India.

Finally, we have not extended the costs derived from this study to their natural progression – a cost–benefit analysis. However, a recent paper on cost–benefit analyses for nutrition interventions indicate that benefit–cost ratio estimates (US$ gains for each US$1 invested) range from 12.9 to 18.4 for Nepal and Bangladesh and 28.9 to 38.6 for Pakistan and India, respectively (Hoddinott *et al.* 2013). At the same time, a recent review on the cost‐effectiveness of nutrition and early childhood interventions highlights the limited availability of cost‐effectiveness studies and notes that even for available studies, comparability of cost‐effectiveness is often limited due to differences in outcomes studied and limited use of common outcome measures across studies (Batura *et al.* 2014).

In conclusion, the need to invest fully for nutrition in India and indeed in all South Asian countries is urgent. This study has estimated the financial commitments required to deliver at scale a set of interventions already within the policy frameworks in India, a country that contributes the largest number of stunted children in the South Asia region. The financial requirements for delivering these interventions vary within India, and prioritization of financing for nutrition across India will need to consider the gaps between projected costs for each state, current expenditures and the availability of national‐level and state‐level finances to deliver fully for nutrition. Further research is essential to re‐estimate some of these costs based on updated unit costs for supplementary feeding, updated target population estimates for severe acute malnutrition and any other updates to interventions and related unit costs.

## Source of funding

POSHAN (Partnerships and Opportunities to Strengthen and Harmonize Actions for Nutrition in India), led by IFPRI, and funded by the Bill and Melinda Gates Foundation.

## Conflicts of interest

The authors declare that they have no conflicts of interest. The authors alone are responsible for the views expressed in this publication and they do not necessarily represent the decisions, policy or views of IFPRI or FEWS NET. Any errors are our own.

## Contributions

PM conceptualized and provided leadership for the study, provided interpretation of data and results and revised the manuscript critically for important intellectual content. CM lead the acquisition and analysis of the data and drafted the manuscript. SC supported data acquisition, analysis, manuscript drafting and revision.

**Abbreviations and Acronyms:**

BCC: Behavioral Change Communication
GOI: Government of India
ICDS: Integrated Child Development Services Program
IEC: Information, Education and Communication
IFA: Iron‐folic acid
IYCF: Infant and Young Child Feeding
NFHS‐III: National Family Health Survey‐III
NRHM: National Rural Health Mission
POSHAN: Partnerships and Opportunities to Strengthen and Harmonize Actions for Nutrition in India
SAM: Severe Acute Malnutrition
SRS: Sample Registration System
SUN: Scaling up Nutrition
SUNWWIC: Scaling up Nutrition: What will it Cost?
UNICEF: United Nations Children's Fund
WAZ: Weight‐for‐Age Z score
WHZ: Weight‐for‐Height Z score
NSS: National Sample Survey
US$: United States Dollar
INR: Indian National Rupee
